# Hypericin-Mediated Antimicrobial Photodynamic Therapy in Dentistry: A Systematic Review of Applications Against Oral Biofilms and Infections

**DOI:** 10.3390/pharmaceutics18040491

**Published:** 2026-04-16

**Authors:** Radosław Turski, Maciej Dobrzyński, Aleksandra Warakomska, Magdalena Pietrzko, Iwona Gregorczyk-Maga, Dariusz Skaba, Rafał Wiench

**Affiliations:** 1 Department of Periodontal and Oral Mucosa Diseases, Faculty of Medical Sciences in Zabrze, Medical University of Silesia, 40-055 Katowice, Poland; awarakomska@sum.edu.pl (A.W.); rwiench@sum.edu.pl (R.W.); 2 Department of Pediatric Dentistry and Preclinical Dentistry, Wroclaw Medical University, 31-155 Kraków, Poland; maciej.dobrzynski@umw.edu.pl; 3 Privat Dental Office Stomatologia Pietrzko, 43-300 Bielsko-Biała, Poland; pietrzkoperio@gmail.com; 4 Department of Developmental Age Dentistry, Institute of Dentistry, Jagiellonian University Medical College, 31-155 Krakow, Poland; iwona.gregorczyk-maga@uj.edu.pl

**Keywords:** hypericin, antimicrobial photodynamic therapy, oral biofilm, dentistry, photosensitizer, periodontal infections, endodontic disinfection, oral candidiasis

## Abstract

**Background:** Oral biofilms are a major etiological factor in dental caries, periodontal disease, peri-implantitis, and endodontic infections. Increasing antimicrobial resistance and the limitations of conventional therapies have intensified interest in antimicrobial photodynamic therapy (aPDT). Hypericin, a natural photosensitizer derived from *Hypericum perforatum*, demonstrates potent reactive oxygen species generation and broad antimicrobial activity; however, its dental applications remain insufficiently synthesized. **Objective:** To systematically evaluate the antimicrobial efficacy, treatment parameters, safety, and clinical potential of hypericin-mediated aPDT against oral biofilms and infections in dentistry. **Methods:** This systematic review was conducted according to PRISMA 2020 and registered in PROSPERO CRD42024617727. Electronic searches of PubMed/MEDLINE, Embase, Scopus, and the Cochrane Library (January 2010 to December 2025) were performed. Studies assessing hypericin-mediated aPDT in oral or dental contexts were included. Methodological quality was evaluated using a predefined nine-domain risk-of-bias tool. **Results:** Eleven studies met the inclusion criteria. Hypericin-mediated aPDT demonstrated strong antimicrobial effects, achieving up to 99% planktonic inactivation and significant biofilm reduction across bacterial and fungal species. Activity was particularly pronounced against Gram-positive organisms, including *Staphylococcus aureus* and *Enterococcus faecalis*. However, efficacy against mature biofilms was variable and often dependent on formulation and irradiation parameters. Most studies showed moderate methodological quality, with frequent deficiencies in reporting light calibration and dosimetry. Advanced delivery systems, including liposomal and nanoparticle formulations, improved photodynamic performance. **Conclusions:** Hypericin-mediated aPDT shows promising antimicrobial activity against oral pathogens and biofilms, with favorable selectivity and safety profiles. Nevertheless, the evidence remains predominantly preclinical and heterogeneous. Standardized protocols and well-designed clinical trials are required before routine dental implementation can be recommended.

## 1. Introduction

Oral biofilms constitute a major and persistent global health burden, responsible for up to 80% of bacterial and fungal infections in humans and representing the principal etiological driver of dental caries, periodontal disease, peri-implantitis, and endodontic infections [1]. These structured microbial communities, enclosed within an extracellular polymeric matrix, display pronounced tolerance to conventional antimicrobial agents and mechanical removal, creating substantial challenges for effective clinical control [2]. Importantly, regular oral hygiene practices and professional mechanical plaque removal remain the cornerstone of biofilm control and can substantially reduce the risk and severity of biofilm-associated oral diseases. Therefore, any adjunctive antimicrobial strategy, including aPDT, should be considered within the context of optimized mechanical debridement rather than as a standalone alternative [1,2]. The increasing prevalence of antibiotic-resistant organisms and the shortcomings of standard therapeutic strategies have further reinforced the demand for novel interventions capable of eradicating pathogenic biofilms while maintaining the ecological stability of the oral microbiome [3,4]. Antimicrobial photodynamic therapy (aPDT) has gained recognition as a non-invasive therapeutic option for oral infectious diseases, based on the combined action of a photosensitizer, visible light, and oxygen to generate reactive oxygen species (ROS) that induce microbial cell death [1,5]. In contrast to antibiotics, aPDT provides several key advantages: rapid and broad-spectrum microbial killing without promoting resistance, activity against both planktonic microorganisms and established biofilms, and the ability to localize treatment with minimal systemic exposure [2,6]. Clinical investigations have reported reductions exceeding 99.9% in early oral biofilm viability and approximately 95% in mature biofilms, alongside measurable shifts in microbial composition [7].

Hypericin, a naturally derived naphthodianthrone compound obtained from St. John’s wort (*Hypericum perforatum* L.), has attracted significant interest as a highly effective photosensitizer for antimicrobial use [8,9]. It exhibits strong absorption in the visible spectrum, efficient production of singlet oxygen and other ROS after photoactivation, and selective accumulation within microbial cells and neoplastic tissues [10,11]. Hypericin demonstrates notable potency against Gram-positive bacteria, with minimum bactericidal concentrations reported between 0.625 and 10 μM and reductions greater than 6 log in methicillin-resistant *Staphylococcus aureus* (MRSA) populations [12,13]. Its antimicrobial action is mediated through ROS-induced damage to multiple cellular structures, including proteins, lipids, and nucleic acids, resulting in irreversible disruption of cell walls, membranes, and intracellular components [11,12,13,14].

Nevertheless, despite strong in vitro findings and efficacy in wound infection models, clinical translation of hypericin has been constrained by poor aqueous solubility, limited bioavailability, and relatively high production costs [8,9]. Recent progress in formulation science, such as liposomal delivery, cyclodextrin complexes, and nanoparticle-based carriers, has shown potential to mitigate these limitations and improve hypericin’s photodynamic performance [8,15]. Moreover, although photosensitizers like methylene blue, toluidine blue, and chlorin e6 have been widely investigated in dental contexts, hypericin’s distinct photophysical profile and natural origin make it an appealing candidate for oral biofilm-targeted therapy [5,16].

The use of hypericin-mediated aPDT in dentistry remains insufficiently explored, with scarce systematic assessment of its antimicrobial effectiveness, optimal irradiation parameters, and clinical outcomes across different oral infectious diseases. Current dental evidence on aPDT largely centers on synthetic photosensitizers, and standardized protocols for hypericin-based applications are lacking [5]. Important uncertainties persist regarding ideal hypericin concentrations, light dosimetry, exposure duration, and possible synergistic effects when combined with conventional dental treatment modalities.

This systematic review aims to comprehensively evaluate the current evidence on hypericin-mediated antimicrobial photodynamic therapy for treating oral biofilms and infections. Specifically, we seek to: (1) assess the antimicrobial efficacy of hypericin-PDT against key oral pathogens and biofilm communities; (2) identify optimal treatment parameters including photosensitizer concentration, light wavelength, energy density, and exposure time; (3) evaluate clinical applications across different dental infectious diseases including periodontal disease, peri-implantitis, endodontic infections, and dental caries; (4) examine the safety profile and potential adverse effects of hypericin-PDT in oral tissues; (5) identify knowledge gaps and future research directions for translating hypericin-mediated aPDT into routine dental practice. By synthesizing available evidence, this review aims to provide clinicians and researchers with a comprehensive understanding of hypericin’s potential role in modern dental antimicrobial therapy and guide future clinical investigations in this promising field.

## 2. Materials and Methods

### 2.1. Focused Question

This systematic review was conducted according to the PICO framework, defined as follows: In patients or experimental models with oral infections and biofilm-associated microbial colonization in dentistry (Population), does treatment with hypericin-mediated antimicrobial photodynamic therapy (Intervention), compared with light irradiation alone, hypericin application without activation, conventional antimicrobial strategies, or other adjunctive disinfection methods (Comparison), result in greater microbial eradication, biofilm reduction, or clinical improvement of oral infectious outcomes (Outcome)? [17].

### 2.2. Search Strategy

**Figure 1:**
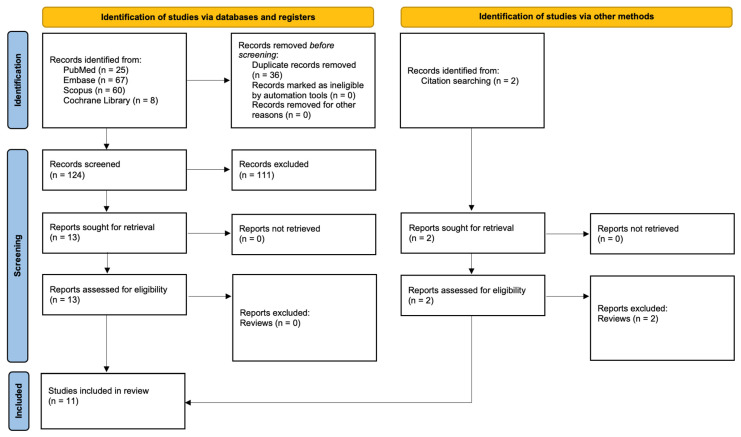
PRISMA 2020 Flowchart.

This systematic review was registered in the PROSPERO database (ID: CRD42024617727) and conducted in accordance with the Preferred Reporting Items for Systematic Reviews and Meta-Analyses (PRISMA) 2020 guidelines [18] (Table S1). An electronic literature search was performed in PubMed/MEDLINE, Embase, Scopus, and the Cochrane Library (full search strategy presented in Figure 1).

Three authors independently performed the database searches using identical search terms (Table 1). Filters were applied to include only articles published in English between 1 January 2010 and 3 December 2025. Following the initial screening, potentially eligible studies were selected based on titles and abstracts according to predefined inclusion criteria. Full-text assessment was subsequently conducted by two reviewers to confirm eligibility and extract relevant data. In addition, a snowball search was performed by manually screening the reference lists of all included full-text articles to identify further relevant publications.

The selection of PubMed/MEDLINE, Embase, Scopus, and the Cochrane Library was based on their complementary coverage of biomedical, clinical, and interdisciplinary literature, including dentistry and photodynamic therapy research.

### 2.3. Selection of Studies

During study selection, reviewers independently evaluated titles and abstracts to minimize selection bias. Any disagreements regarding eligibility were resolved through structured discussion until consensus was achieved. This methodology, consistent with PRISMA standards, ensured that only relevant and methodologically appropriate studies were included, thereby improving the reliability and reproducibility of the review [19].

### 2.4. Methodological Reporting Assessment

To reduce potential bias in study inclusion, reviewers independently assessed titles, abstracts, and full texts. Inter-reviewer agreement was quantified using Cohen’s kappa statistic. Discrepancies were resolved through discussion among the authors until a unanimous agreement was reached [19].

The methodological quality of included studies was assessed using a predefined nine-domain reporting checklist focusing on key parameters relevant to antimicrobial photodynamic therapy, including photosensitizer characterization, incubation protocols, light dosimetry, control conditions, and outcome reporting. Given the predominance of in vitro and preclinical study designs, a formal study-design-specific risk-of-bias tool (e.g., RoB 2 or ROBINS-I) was not applicable. Instead, this approach aimed to evaluate the completeness and transparency of methodological reporting rather than internal validity per se. Risk of bias was evaluated using a predefined nine-domain scoring tool, with each criterion scored as 1 (“yes”) or 0 (“no”):

1. Was the concentration of hypericin as the photosensitizer clearly reported?
2. Was the origin or formulation of hypericin specified?
3. Was the incubation time prior to irradiation stated?
4. Were light source parameters (wavelength, output power, fluence, irradiance) adequately described?
5. Was light output calculated using a power meter?
6. Was an appropriate negative control group included?
7. Were quantitative outcomes reported with relevant statistical analysis?
8. Was outcome reporting complete, with no missing data?
9. Was the study free from evident funding-related conflicts?

Studies were categorized according to total scores as follows: high risk of bias (0–3), moderate risk (4–6), and low risk (7–9). Classification was performed in accordance with principles outlined in the Cochrane Handbook for Systematic Reviews of Interventions [20].

The overall methodological quality and risk of bias across the included studies are summarized in Section 3.3.

### 2.5. Data Extraction

After final consensus on study inclusion, two reviewers extracted data using a standardized approach. Extracted variables included: author and year, study type, microbial species investigated, experimental and control conditions, follow-up duration (if applicable), primary outcomes, hypericin concentration, incubation and irradiation protocols, light source characteristics, and the use of delivery systems such as nanocarriers or adjunctive compounds.

## 3. Results

### 3.1. Study Selection

The PRISMA flow diagram illustrates the study selection process. A total of 160 records were initially identified through database searching, including PubMed (*n* = 25), Embase (*n* = 67), Scopus (*n* = 60), and the Cochrane Library (*n* = 8). After removal of 36 duplicates, 124 records underwent title and abstract screening, of which 111 were excluded. Thirteen reports were sought for retrieval, and all were successfully obtained and assessed for eligibility, with none excluded at this stage. Additionally, two records were identified through citation searching; both were retrieved and assessed but subsequently excluded as review articles. Ultimately, 11 studies met the eligibility criteria and were included in the final review.

### 3.2. Quality Assessment

The methodological quality of the included studies was generally moderate to good, with total scores ranging from 4 to 7 out of 9. Across studies, reporting of hypericin concentration, incubation time, inclusion of negative controls, quantitative outcome reporting, and completeness of data were consistently adequate. However, a recurrent methodological limitation was insufficient reporting of detailed light source parameters and a lack of explicit calibration of light output using a power meter. Additionally, none of the studies clearly demonstrated the absence of funding-related conflicts within the assessed domain. Overall, while the body of evidence shows acceptable experimental reporting in key areas, improvements in photophysical parameter standardization and transparency of methodological details remain necessary to reduce the risk of bias in antimicrobial photodynamic therapy research. Table 2 shows a summary of this assessment.

### 3.3. Main Outcomes

Across the included studies, hypericin-mediated antimicrobial photodynamic therapy demonstrated broad antimicrobial and antibiofilm activity in diverse experimental models. Amaral et al. (2020) reported up to 99% planktonic inactivation of *Enterococcus faecalis* with approximately 60% biofilm reduction and no cytotoxicity toward fibroblasts, indicating selective antimicrobial action [21]. Fernandes et al. (2023) showed significant growth reduction in 75% of *Trichophyton rubrum* isolates, with complete inhibition in 56.25% and confirmed efficacy against azole-resistant strains [22]. García et al. (2015) observed a clear bactericidal effect against MSSA and MRSA in planktonic cultures, although biofilms required longer incubation and efficacy correlated with biofilm-forming capacity [23]. Kashef et al. (2015) found hypericin alone ineffective against *Staphylococcus aureus* biofilms, whereas the combination with acetylcysteine achieved 5.2 to 6.3 log reduction and approximately 6.5 log killing in planktonic cells [24]. Malacrida et al. (2020) demonstrated about 4 log CFU reduction in planktonic *S. aureus* and approximately 0.9 log reduction in biofilm, with improved outcomes after prolonged illumination [25]. Nafee et al. (2013) confirmed enhanced antibiofilm activity against MRSA using nano-hypericin, achieving up to 90 to 100% inhibition and improved wound healing in vivo [26]. Mechanistic cellular studies by Olek et al. showed dose-dependent phototoxicity in SCC-25 oral cancer cells with selective immunomodulatory effects, including increased sTNF-R1 and modulation of sIL-6Rβ, IL-20, IL-8, IL-32, and Pentraxin-3 [27,28]. Plenagl et al. (2019) reported up to 4.1 log reduction in *Staphylococcus saprophyticus* using liposomal formulations, with complete eradication using inclusion complexes [29]. Sakita et al. (2019) demonstrated approximately 70% complete inhibition of *Candida* spp. and synergism with fluconazole [30]. Finally, Vollmer et al. (2019) showed complete eradication of initial in situ oral biofilms and substantial reduction in mature biofilms, accompanied by shifts in microbial composition [31]. Table 3 is a summary of information from these studies.

## 4. Discussion

### 4.1. Principal Findings and Clinical Implications

This systematic review evaluated the current evidence on hypericin-mediated antimicrobial photodynamic therapy for treating oral biofilms and infections in dentistry. However, the evidence base remains limited compared to conventional photosensitizers, and significant heterogeneity in treatment protocols presents challenges for clinical translation. The antimicrobial effectiveness of hypericin-PDT against oral biofilms appears robust based on available in vitro and ex vivo studies. Vollmer et al. demonstrated that *Hypericum perforatum* extract containing hypericin at 32 mg/mL, combined with visible light plus water-filtered infrared-A (VIS + wIRA), achieved complete eradication of initial oral biofilms (100%) and substantial reduction in mature biofilms (>92% when rinsed, 13% when not rinsed prior to activation) in an in situ model [32]. Although hypericin-mediated aPDT has been shown to alter microbial diversity and richness within oral biofilms, the clinical relevance of these changes remains uncertain. Such shifts are not inherently beneficial and may reflect ecological disruption rather than selective suppression of pathogenic taxa [33].

Despite the promising antimicrobial effects observed for hypericin-mediated aPDT, it is essential to emphasize that mechanical biofilm disruption through oral hygiene measures and professional debridement remains the primary and most evidence-based strategy for managing oral biofilm-associated diseases. The structural organization of biofilms limits the penetration and efficacy of antimicrobial agents, including photosensitizers, making physical disruption a prerequisite for effective treatment. Consequently, hypericin-PDT should be interpreted as a potential adjunct rather than a replacement for conventional mechanical approaches. Future studies should therefore investigate combined protocols, directly comparing mechanical debridement alone versus debridement supplemented with hypericin-mediated aPDT to determine any clinically meaningful additive benefit.

### 4.2. Antimicrobial Spectrum and Mechanistic Considerations

Hypericin demonstrates particularly robust activity against Gram-positive bacteria, which constitute important periodontal pathogens, including *Streptococcus* species and *Actinomyces* [34,35]. The photosensitizer’s mechanism involves the generation of reactive oxygen species (ROS) upon photoactivation, leading to multi-target cellular damage, including disruption of bacterial membranes, oxidation of proteins and lipids, and damage to nucleic acids [36,37]. This multi-target mechanism theoretically reduces the likelihood of resistance development, a critical advantage over conventional antibiotics in the context of increasing antimicrobial resistance [38,39]. The efficacy of hypericin-PDT extends beyond bacteria to include antifungal activity. A recent systematic review by Łopaciński et al. found that hypericin-mediated aPDT achieved significant reductions in *Candida* biofilm and planktonic cell viability, including fluconazole-resistant strains, with minimal cytotoxicity to host tissues [39]. This broad-spectrum antimicrobial activity suggests that hypericin may be a candidate for further investigation in polymicrobial oral infection models, which are characteristic of periodontal disease and peri-implantitis.

A critical finding of this review is the lack of standardized treatment protocols for hypericin-PDT in dentistry. The optimal hypericin concentration, light wavelength, energy density, and exposure time remain poorly defined for oral applications. Studies in dermatology have employed hypericin concentrations ranging from 0.25% topically with light doses of 5 to 12 J/cm^2^ at wavelengths of 500 to 650 nm [40,41,42]. However, direct extrapolation to the oral cavity is problematic given differences in tissue architecture, biofilm characteristics, and accessibility.

The wavelength-dependent properties of hypericin-PDT warrant particular attention. Hypericin exhibits strong absorption peaks in the visible light spectrum, with maximum absorption around 590 nm [43]. Studies have demonstrated that longer wavelengths (590 nm) achieve greater tissue penetration depth compared to shorter wavelengths (550 nm), though the relationship is complex and influenced by vascular effects beyond simple photophysical considerations [43]. For dental applications, the choice of wavelength must balance photosensitizer activation efficiency with adequate penetration into periodontal pockets, dentinal tubules, or biofilm structures.

The pre-irradiation incubation time represents another critical parameter requiring optimization. Hypericin demonstrates preferential accumulation in microbial cells and neoplastic tissues, with peak cellular uptake typically occurring within 2 to 24 h, depending on the delivery system [44,45]. For oral biofilm applications, shorter incubation times (2 to 5 min) have been employed with *Hypericum perforatum* extracts, though whether this allows sufficient photosensitizer penetration into mature biofilms remains uncertain [32].

### 4.3. Delivery System Innovations and Formulation Challenges

The poor water solubility and low bioavailability of hypericin represent major obstacles to clinical implementation [34,46]. These physicochemical limitations have prompted the development of various delivery systems to enhance hypericin’s therapeutic potential. Liposomal encapsulation of hypericin-cyclodextrin inclusion complexes has shown promise, achieving 4.1 log reduction in *Staphylococcus* growth in planktonic cultures and effective biofilm treatment in vitro [34]. Similarly, PEGylated mesoporous silica nanoparticles loaded with hypericin demonstrated enhanced ROS generation and superior photodynamic antibacterial activity compared to free hypericin [47]. Thermoresponsive liposomes co-loaded with hypericin-cyclodextrin complexes and near-infrared dyes represent an innovative approach, enabling photo-triggered release and synergistic photothermal-photodynamic therapy [48]. Such dual-mechanism systems achieved >4 log reduction in bacterial viability, suggesting potential for enhanced antimicrobial effects under experimental conditions. However, these advanced delivery systems remain in preclinical development, and their translation to clinical dentistry requires addressing manufacturing complexity, cost considerations, and regulatory pathways. The use of whole *Hypericum perforatum* extracts rather than purified hypericin presents both advantages and challenges. Extracts contain multiple bioactive compounds that may exhibit synergistic antimicrobial effects or provide protective effects against phototoxicity [32,49,50]. However, batch-to-batch variability in hypericin content and the presence of other photosensitizing compounds complicate dose standardization and reproducibility.

### 4.4. Comparison with Established Photosensitizers

When compared to conventional photosensitizers used in dental aPDT, hypericin exhibits distinct advantages and limitations. Methylene blue and toluidine blue remain the most extensively studied photosensitizers for periodontal applications, with well-established clinical protocols and demonstrated efficacy in reducing periodontal pathogens and improving clinical parameters [51,52]. A recent meta-analysis found that toluidine blue was the most effective photosensitizer for peri-implantitis treatment, showing superior improvements in bleeding on probing, probing depth, and crestal bone loss [52].

Hypericin offers several theoretical advantages over these synthetic dyes, including its ROS generation capacity and preferential accumulation in target cells [45,53]. Comparative in vitro studies have shown that hypericin induces high phototoxicity at very low concentrations and is particularly effective in inducing apoptosis over a wide range of light fluences [53]. However, the clinical superiority of hypericin over established photosensitizers has not been demonstrated in head-to-head trials in dental applications. The absorption spectrum of hypericin (peak around 590 nm) differs from that of methylene blue (660 nm) and toluidine blue (630 nm), potentially affecting tissue penetration depth [43,51]. While longer wavelengths generally penetrate deeper into tissues, hypericin’s efficient ROS generation and multi-target mechanism may compensate for this limitation in superficial oral applications.

### 4.5. Clinical Applications in Periodontal Disease

The application of aPDT as an adjunct to scaling and root planing (SRP) for periodontal disease has been extensively investigated with various photosensitizers [54,55,56]. While hypericin-specific clinical trials in periodontology are limited, the broader aPDT literature provides indirect context for hypothesis generation. Systematic reviews have demonstrated that aPDT, adjunctive to SRP, significantly improves clinical parameters, including probing depth reduction, clinical attachment gain, and bleeding on probing compared to SRP alone [54,56].

A recent randomized clinical trial using photodynamic therapy (not hypericin-specific) reported significant improvements in clinical parameters, with 73.3% reduction in bleeding on probing, a 1.9 mm reduction in probing depth, and 0.6 mm clinical attachment gain, along with a significant reduction in red complex bacteria [55]. It remains unknown whether hypericin-PDT can achieve comparable outcomes to established photosensitizers in this indication. The antimicrobial selectivity of aPDT represents an important consideration for periodontal applications. While broad-spectrum microbial killing may be desirable for acute infections, selective targeting of pathogenic species while preserving commensal flora would be optimal for long-term periodontal health [57]. Whether hypericin exhibits sufficient selectivity for periodontal pathogens over beneficial oral microbiota requires further investigation.

### 4.6. Endodontic Applications and Root Canal Disinfection

Photodynamic therapy has emerged as a promising adjunct for endodontic disinfection, particularly for persistent infections resistant to conventional chemomechanical preparation [58]. The complex anatomy of root canal systems, including dentinal tubules, isthmuses, and apical ramifications, creates sanctuaries for bacterial biofilms that are difficult to eliminate with mechanical instrumentation and irrigation alone.

Hypericin’s photophysical properties may warrant investigation in endodontic applications. Its preferential accumulation in bacterial cells and efficient ROS generation at low concentrations could potentially contribute to antimicrobial effects with minimal tissue damage [36,45]. However, light delivery into root canal systems presents technical challenges, requiring specialized fiber-optic delivery systems and consideration of light scattering and absorption by dentin.

The depth of antimicrobial effect achievable with hypericin-PDT is particularly relevant for endodontics. Studies in tumor models have demonstrated that hypericin-PDT can achieve tissue effects at depths of 7.5 to 9.9 mm, depending on wavelength and light dose, suggesting potential for adequate penetration into dentinal tubules [43]. However, the optical properties of dentin differ substantially from soft tissues, and specific studies in endodontic models are needed to validate this application.

### 4.7. Safety Profile and Tissue Biocompatibility

The safety profile of hypericin-PDT appears favorable based on available evidence. Clinical trials in dermatology have reported that adverse events are primarily mild, self-limited skin reactions including pruritus, hyperpigmentation, burning, and application-site irritation, with severe treatment-related adverse events occurring in less than 2% of patients [41]. Importantly, hypericin showed no evidence of systemic absorption following topical application, suggesting minimal risk of systemic toxicity [41]. The photosensitization risk associated with hypericin deserves careful consideration. While high-dose oral administration of *Hypericum perforatum* extracts can increase cutaneous photosensitivity in humans, the effect is generally mild and clinically manageable [49,50]. Studies have shown that typical clinical doses do not provide evidence for significant phototoxic potential in humans [50]. For topical dental applications with localized light exposure, the risk of generalized photosensitization would be expected to be minimal. Cytotoxicity studies have demonstrated that hypericin exhibits minimal dark toxicity to mammalian cells, with phototoxic effects being highly selective for target cells upon light activation [36,59]. This selectivity is crucial for dental applications where preservation of healthy periodontal tissues, gingival fibroblasts, and pulp vitality is essential. Studies in oral cancer cells have shown that hypericin-PDT can modulate inflammatory cytokine secretion, potentially contributing to tissue healing beyond direct antimicrobial effects [59].

### 4.8. Limitations and Knowledge Gaps

Several critical knowledge gaps limit the current clinical application of hypericin-PDT in dentistry. First, the absence of randomized controlled clinical trials specifically evaluating hypericin-mediated aPDT for dental indications represents a major limitation. While in vitro and ex vivo studies demonstrate promising antimicrobial activity, clinical efficacy, optimal treatment protocols, and long-term outcomes remain undefined. The heterogeneity in hypericin formulations, concentrations, and delivery systems across studies precludes meaningful meta-analysis and evidence synthesis. Standardization of hypericin preparations is essential for reproducible research and clinical translation. The pharmacokinetics and tissue distribution of hypericin in oral tissues have not been adequately characterized. Understanding penetration into periodontal pockets, biofilm matrices, and dentinal tubules is crucial. The long-term effects of repeated hypericin-PDT treatments on the oral microbiome require investigation. Although the multi-target mechanism of PDT theoretically prevents resistance development, ecological consequences must be clarified. The cost-effectiveness analyses comparing hypericin-PDT to conventional treatments are lacking. The higher production costs of hypericin and the need for specialized light delivery systems may limit accessibility in routine dental practice [34,46].

Several research priorities emerge from this systematic review. Well-designed randomized controlled trials comparing hypericin-PDT with established photosensitizers are urgently needed. Optimization studies defining ideal treatment parameters should be prioritized. Development and validation of improved delivery systems remain critical translational goals [34,47]. Mechanistic studies on biofilm architecture and host responses are also warranted. Finally, robust economic analyses are needed to inform healthcare policy and reimbursement decisions [59].

### 4.9. Clinical Translation Considerations

For successful translation of hypericin-PDT into routine dental practice, several practical considerations must be addressed. Standardized treatment protocols with clear indications, contraindications, and step-by-step procedures are essential for clinician adoption. Development of user-friendly delivery systems and light sources specifically designed for dental applications would facilitate implementation. Patient education regarding the natural origin of hypericin, the mechanism of photodynamic therapy, and expected outcomes may enhance acceptance and compliance. The need for light protection following treatment, if any, should be clearly communicated, though topical dental applications would be expected to pose minimal photosensitization risk. Regulatory pathways for hypericin-based dental products require clarification. Whether hypericin formulations would be classified as drugs, medical devices, or combination products varies by jurisdiction and formulation, affecting the regulatory requirements for market approval. Training and education of dental professionals in aPDT principles, hypericin-specific considerations, and safe light delivery techniques will be necessary for widespread adoption. Integration of aPDT training into dental curricula and continuing education programs can facilitate knowledge dissemination and skill development.

Compared with conventional antimicrobial strategies, including mechanical debridement, antiseptics such as chlorhexidine, and systemic or local antibiotics, hypericin-mediated aPDT demonstrates comparable antimicrobial activity under controlled experimental conditions, particularly against planktonic microorganisms and early-stage biofilms. However, its efficacy against mature, structured biofilms remains variable and generally inferior to the combined effect of mechanical disruption and chemical disinfection. In contrast to antibiotics, aPDT exerts a multi-target oxidative mechanism of action, which is associated with a low likelihood of inducing microbial resistance. From a safety perspective, available evidence suggests minimal dark toxicity and selective phototoxicity toward microbial cells, although these findings are largely derived from in vitro models and non-oral clinical contexts. Importantly, unlike conventional therapies, hypericin-based aPDT lacks robust clinical data demonstrating superiority or even equivalence in dental applications. Therefore, at present, it should be regarded as a potential adjunct rather than an alternative to established antimicrobial approaches.

The translation of hypericin-mediated aPDT from preclinical models to standardized clinical practice is limited by several critical factors. First, substantial heterogeneity exists in experimental protocols, including variations in hypericin concentration, formulation, incubation time, light wavelength, fluence, and irradiation duration, which preclude direct comparison between studies and prevent protocol standardization. Second, most available evidence is derived from simplified in vitro or ex vivo models that do not adequately replicate the structural complexity of oral biofilms, host immune responses, or the dynamic oral environment. Third, hypericin’s physicochemical limitations, including poor aqueous solubility and variable bioavailability, complicate its delivery and reproducibility in clinical settings. Finally, the absence of randomized controlled clinical trials in dental indications represents a major barrier to evidence-based implementation, as efficacy, safety, and long-term outcomes in patients remain undefined.

The variable effectiveness of hypericin-mediated aPDT against mature biofilms highlights the need for optimization of both formulation and irradiation parameters. From a formulation perspective, strategies that enhance photosensitizer penetration into the extracellular polymeric matrix, such as encapsulation in nanocarriers or the use of biofilm-disrupting adjuncts (e.g., surfactants or mucolytic agents), may improve antimicrobial outcomes. From a photophysical standpoint, optimization of light dosimetry, including appropriate wavelength selection aligned with hypericin absorption peaks, increased fluence, and adequate irradiation time, is essential to achieve sufficient reactive oxygen species generation within deeper biofilm layers. In addition, sequential or combined approaches integrating mechanical biofilm disruption with aPDT may be necessary, as physical removal of the biofilm matrix appears to be a prerequisite for effective photodynamic action in complex, mature biofilms.

Advanced delivery systems, including liposomal, polymeric, and nanoparticle-based formulations, play a significant role in enhancing the photodynamic performance of hypericin. These systems improve aqueous solubility, facilitate targeted delivery, and increase intracellular accumulation of the photosensitizer, thereby enhancing reactive oxygen species generation and antimicrobial efficacy. Several studies included in this review demonstrated improved outcomes using such formulations compared with free hypericin, particularly in biofilm models. However, these approaches remain largely experimental and are associated with important limitations, including increased manufacturing complexity, potential variability in physicochemical properties, challenges in large-scale production, and regulatory uncertainty. Furthermore, their safety and pharmacokinetics in oral tissues have not been sufficiently characterized, which currently limits their clinical applicability.

Future research should prioritize well-designed randomized controlled clinical trials evaluating hypericin-mediated aPDT in specific dental indications, including periodontal disease, peri-implantitis, and endodontic infections, with direct comparison to standard-of-care treatments. Standardization of treatment protocols is essential and should include clearly defined parameters for photosensitizer concentration, formulation, incubation time, light wavelength, fluence, and irradiation duration. In addition, studies should investigate combined treatment strategies integrating mechanical debridement with aPDT to determine potential additive or synergistic effects. Long-term evaluations of clinical outcomes, microbiological shifts, and oral microbiome stability are also required to assess both efficacy and ecological impact. Finally, further research into optimized delivery systems and cost-effectiveness analyses will be necessary to determine the feasibility of implementing hypericin-mediated aPDT in routine dental practice.

## 5. Conclusions

Hypericin-mediated antimicrobial photodynamic therapy demonstrates substantial antimicrobial potential against key oral pathogens and biofilm communities relevant to dentistry. The available evidence indicates strong activity against planktonic microorganisms and moderate but variable efficacy against mature biofilms, particularly when advanced delivery systems such as liposomal or nanoparticle formulations are employed. The therapy shows favorable selectivity with minimal cytotoxicity toward host cells, supporting its biological plausibility as an adjunctive antimicrobial strategy. However, the current evidence base remains limited and predominantly preclinical. Considerable heterogeneity exists in hypericin formulations, concentrations, incubation protocols, and light dosimetry, which prevents direct comparison between studies and precludes firm clinical recommendations. In addition, the lack of randomized controlled clinical trials in periodontal, peri-implant, and endodontic settings represents a major translational gap. At present, hypericin-mediated aPDT should be regarded as a promising but investigational modality in dental infection control. Future research must prioritize standardized photodynamic protocols, rigorous in vivo and clinical studies, detailed photophysical reporting, and long-term assessments of microbiological and clinical outcomes. Addressing these gaps will be essential to determine whether hypericin can achieve a clinically meaningful role alongside established photosensitizers in evidence-based dental practice.

## Figures and Tables

**Table 1:** Search syntax used in the study.

Source	Search Syntax	N
PubMed	(“Hypericin” [Mesh] OR hypericin [tiab] OR hypericum [tiab]) AND (“Photochemotherapy” [Mesh] OR “photodynamic therapy” [tiab] OR PDT [tiab] OR aPDT [tiab] OR “antimicrobial photodynamic” [tiab]) AND (“Dentistry” [Mesh] OR “Mouth” [Mesh] OR “Oral Health” [Mesh] OR dent* [tiab] OR oral [tiab] OR periodont* [tiab] OR endodont* [tiab] OR caries [tiab] OR biofilm [tiab])	25
Embase	(‘hypericin’/exp OR hypericin:ti,ab OR hypericum:ti,ab) AND (‘photodynamic therapy’/exp OR ‘photodynamic therapy’:ti,ab OR pdt:ti,ab OR apdt:ti,ab OR ‘antimicrobial photodynamic therapy’:ti,ab OR ‘photochemotherapy’/exp) AND (‘dentistry’/exp OR ‘oral health’/exp OR ‘mouth’/exp OR dent*:ti,ab OR oral:ti,ab OR periodont*:ti,ab OR endodont*:ti,ab OR caries:ti,ab OR biofilm:ti,ab OR candid*:ti,ab)	67
Scopus	TITLE-ABS-KEY (hypericin OR hypericum) AND TITLE-ABS-KEY (“photodynamic therapy” OR PDT OR aPDT OR “antimicrobial photodynamic therapy” OR photochemotherapy) AND TITLE-ABS-KEY (dent* OR oral OR periodont* OR endodont* OR caries OR biofilm OR candid*)	60
Cochrane Libary	(hypericin OR hypericum) AND (“photodynamic therapy” OR PDT OR aPDT OR “antimicrobial photodynamic therapy” OR photochemotherapy) AND (dent* OR dentistry OR oral OR “oral health” OR periodont* OR endodont* OR caries OR biofilm OR candid*)	8

**Table 2:** Results of the risk of bias assessment.

Study	1	2	3	4	5	6	7	8	9	Total	RoB Category
Amaral et al., 2020 [21]	1	0	1	0	0	1	1	1	0	5	Moderate
Fernandes et al., 2023 [22]	1	1	1	0	0	1	1	1	0	6	Moderate
García et al., 2015 [23]	1	1	1	0	1	1	1	1	0	7	Low
Kashef et al., 2015 [24]	1	0	0	0	0	1	1	1	0	5	Moderate
Malacrida et al., 2020 [25]	1	1	1	0	1	1	1	1	0	7	Low
Nafee et al., 2013 [26]	1	1	1	0	1	1	1	1	0	7	Low
Olek et al., 2023 [27]	1	0	1	0	0	0	1	1	0	4	Moderate
Olek et al., 2024 [28]	1	0	1	0	0	0	1	1	0	4	Moderate
Plenagl et al., 2019 [29]	1	1	1	0	1	1	1	1	0	7	Low
Sakita et al., 2019 [30]	1	1	1	0	1	0	1	1	0	6	Moderate
Vollmer et al., 2019 [31]	1	1	1	0	1	1	1	1	0	7	Low

**Table 3:** Summary of main findings.

Study	Country	Groups	Microorganisms	Main Outcomes
Amaral et al., 2020 [21]	Brazil	In vitro: planktonic, biofilm, fibroblasts	*Enterococcus faecalis*	Up to 99% planktonic inactivation; ~60% biofilm reduction; no fibroblast cytotoxicity; selective antimicrobial effect.
Fernandes et al., 2023 [22]	Brazil	Planktonic cells; adhesion and mature biofilm	*Trichophyton rubrum*	Significant growth reduction in 75% isolates; 56.25% complete inhibition; effective against azole-resistant strains; biofilm inactivation observed.
García et al., 2015 [23]	Spain	Planktonic vs biofilm	MSSA, MRSA	Bactericidal effect in planktonic cells; longer incubation required for biofilms; activity correlated with biofilm production.
Kashef et al., 2015 [24]	Iran	HYP alone vs HYP + acetylcysteine; planktonic and biofilm	*Staphylococcus aureus*	HYP alone ineffective on biofilms; combination achieved 5.2 to 6.3 log reduction; ~6.5 log killing in planktonic cells.
Malacrida et al., 2020 [25]	Brazil	Planktonic vs biofilm; nanoparticle formulation	*Staphylococcus aureus* ATCC 25923	~4 log CFU reduction planktonic; ~0.9 log reduction biofilm; longer illumination improved efficacy.
Nafee et al., 2013 [26]	Egypt	In vitro + in vivo rat wound model; free vs nano-HYP	MRSA	Enhanced antibiofilm activity; up to ~90 to 100% inhibition in some isolates; improved wound healing and epithelialization.
Olek et al., 2023 [27]	Poland	In vitro; SCC-25 OSCC cells and HGF-1 gingival fibroblasts; hypericin (0–1 µM) with visible light PDT vs controls	SCC-25 oral squamous cell carcinoma cells; HGF-1 fibroblasts	Phototoxic effect observed from 5 J/cm^2^ and increased with hypericin concentration. Significant increase in sTNF-R1 secretion in SCC-25 after PDT. No effect on sTNF-R1 in fibroblasts and no effect on sTNF-R2 in either line, indicating selective immunomodulation.
Olek et al., 2024 [28]	Poland	In vitro; SCC-25 OSCC cells and HGF-1 fibroblasts; hypericin-PDT (0–1 µM; 0–20 J/cm^2^) vs controls	SCC-25 oral cancer cells; HGF-1 gingival fibroblasts	Phototoxicity increased with higher hypericin and light dose. HY-PDT modified secretion of sIL-6Rβ, IL-20, and Pentraxin-3 in SCC-25. Hypericin alone increased IL-8. In fibroblasts, HY-PDT affected IL-8 and IL-32 secretion, confirming immunomodulatory activity.
Plenagl et al., 2019 [29]	Germany	Planktonic and biofilm; liposomal formulations	*Staphylococcus saprophyticus*	Up to 4.1-log reduction (liposomes); inclusion complex achieved total eradication; effective also in biofilm.
Sakita et al., 2019 [30]	Brazil	P123-Hyp ± fluconazole; planktonic and biofilm	*Candida* spp.	~70% isolates completely inhibited; synergism with fluconazole; biofilm formation inhibited in all species.
Vollmer et al., 2019 [31]	Germany/Switzerland	In situ oral biofilm in volunteers	Multispecies oral biofilm	100% eradication without rinsing; >92% reduction in initial biofilm; ~13% reduction in mature biofilm; microbial shift observed.

MSSA, methicillin-susceptible *Staphylococcus aureus*; MRSA, methicillin-resistant *Staphylococcus aureus*; HYP, hypericin; nano-HYP, nanoparticle-formulated hypericin; CFU, colony-forming units; ATCC, American Type Culture Collection; SCC-25, human oral squamous cell carcinoma cell line; OSCC, oral squamous cell carcinoma; HGF-1, human gingival fibroblasts; PDT, photodynamic therapy; HY-PDT, hypericin-mediated photodynamic therapy; sTNF-R1, soluble tumor necrosis factor receptor 1; sTNF-R2, soluble tumor necrosis factor receptor 2; sIL-6Rβ, soluble interleukin-6 receptor beta; IL, interleukin; P123-Hyp, Pluronic P123 micelle-loaded hypericin; spp., species.

## Data Availability

All generated data is presented in the manuscript.

## Supplementary Materials

The following supporting information can be downloaded at: https://www.mdpi.com/article/10.3390/pharmaceutics18040491/s1, Table S1: PRISMA Checklist.
